# β-barrel Oligomers as Common Intermediates of Peptides Self-Assembling into Cross-β Aggregates

**DOI:** 10.1038/s41598-018-28649-7

**Published:** 2018-07-09

**Authors:** Yunxiang Sun, Xinwei Ge, Yanting Xing, Bo Wang, Feng Ding

**Affiliations:** Department of Physics and Astronomy, Clemson University, Clemson, SC 29634 USA

## Abstract

Oligomers populated during the early amyloid aggregation process are more toxic than mature fibrils, but pinpointing the exact toxic species among highly dynamic and heterogeneous aggregation intermediates remains a major challenge. β-barrel oligomers, structurally-determined recently for a slow-aggregating peptide derived from αB crystallin, are attractive candidates for exerting amyloid toxicity due to their well-defined structures as therapeutic targets and compatibility to the “amyloid-pore” hypothesis of toxicity. To assess whether β-barrel oligomers are common intermediates to amyloid peptides - a necessary step toward associating β-barrel oligomers with general amyloid cytotoxicity, we computationally studied the oligomerization and fibrillization dynamics of seven well-studied fragments of amyloidogenic proteins with different experimentally-determined aggregation morphologies and cytotoxicity. In our molecular dynamics simulations, β-barrel oligomers were only observed in five peptides self-assembling into the characteristic cross-β aggregates, but not the other two that formed polymorphic β-rich aggregates as reported experimentally. Interestingly, the latter two peptides were previously found nontoxic. Hence, the observed correlation between β-barrel oligomers formation and cytotoxicity supports the hypothesis of β-barrel oligomers as the common toxic intermediates of amyloid aggregation.

## Introduction

Aggregation of proteins and peptides into amyloid fibrils is associated with more than 25 degenerative diseases, including Alzheimer’s disease (AD)^1,2^, Parkinson’s disease (PD)^3,4^, prion conditions^5^ and type-2 diabetes (T2D)^6,7^. Despite the differences in primary, secondary and tertiary structures of precursor proteins, experimental studies using x-ray crystallography, solid-state NMR or cryo-EM have demonstrated that the final amyloid fibrils share a common cross-β core structure with β-strands aligned perpendicular to the fibril axis and multiple β-sheets facing each other^8–10^. Increasing evidence suggests that soluble low molecular weight oligomer intermediates are more cytotoxic than mature fibrils^11,12^. Since not all oligomers are toxic, characterization of these oligomeric intermediates pinpointing the toxic oligomer species are thus crucial for both understanding the pathogenesis and designing therapeutic approaches for the treatment of amyloid diseases.

Based on the structure−function relationship principle where specific functions of proteins and protein complexes are determined by their distinct conformational states, these toxic oligomers of amyloid aggregation are expected to have well-organized structures to execute their pathological functions^13,14^. For instance, many amyloidogenic proteins and peptides can aggregate in the membrane environment by forming pore-like oligomer structures that disrupt the membrane integrity and permeability^15–17^. Using an 11-residue segment from a slow-aggregating αB crystallin, Laganowsky *et al*. identified a stable oligomer formed by six peptides in the shape of a barrel (i.e., β-barrel) by x-ray crystallography^11^. β-barrel is a common protein fold adapted by many solution and transmembrane proteins. β-barrel oligomers formed by a few individual short peptides have been observed in previous computational studies using molecular dynamics^18,19^, replica exchange molecular dynamics (REMD)^20–22^ or Monte Carlo^23^ simulations. Combining experimental characterizations with computational modeling, Do *et al*. showed that the C-terminal fragments of amyloid-β (Aβ) might form similar β-barrel oligomers^14^. The β-barrel structure as a model for small Aβ40/Aβ42 oligomers was also supported by recent hydrogen exchange mass spectrometry^24^ and NMR studies^25^. Aβ40/Aβ42 oligomers with pore-like conformations was also observed in a recent computational study combining coarse-grained and all-atom simulations^26^. These β-barrel oligomers capable of spanning across the lipid bilayer and thus compatible to the “amyloid-pore” hypothesis of amyloid toxicity^15–17^ have been postulated as the early aggregation intermediates exerting toxic effects on cells^11^. However, the isolation and characterization of β-barrel oligomers from highly heterogeneous and dynamic aggregation intermediates is often experimentally challenging. Hence, the connection of β-barrel oligomers with the general amyloid cytotoxicity in amyloid diseases remains to be fully established. It is unclear whether the formation of β-barrel oligomer as intermediates is common and yet specific to the aggregation of toxic amyloid peptides.

Recently, two overlapping 11-residue fragments of the T2D-associated human islet amyloid polypeptide (hIAPP) - located at residues 15–25 and 19–29 (denoted as hIAPP15–25 and hIAPP19–29) - have been experimentally found to display contrasting cytotoxicity while both being able to form β-sheet rich aggregates^27^. The fibrils of hIAPP19–29 with S20G mutation had mated β-sheets (i.e., the cross-β structure) with inter-digit packing of hydrophobic surfaces and were similarly cytotoxic as the full-length hIAPP fibrils, but hIAPP15–29 formed non-toxic labile β-sheet aggregates. S20G is a disease-causing mutation which renders both full-length^28,29^ and fragment hIAPPs (e.g., hIAPP18–29^30^) more aggregation-prone and cytotoxic. With small sizes and distinct aggregation morphologies and cytotoxicity, hIAPP15–25, hIAPP19–29 and their S20G mutants are therefore the ideal model system to investigate the relationship between the propensity to form β-barrel oligomers as aggregation intermediates and amyloid cytotoxicity. In addition, to answer whether other amyloid peptides could also form β-barrel oligomer intermediates, we studied hIAPP22-28^31^, Aβ16–22^32^ and NACore^33^ (residues 68–78 in α-synuclein), corresponding to the amyloidogenic cores of hIAPP, Aβ and α-synuclein implicated in T2D, AD and PD, respectively. All three peptides were documented to form amyloid fibrils and found cytotoxic in experiments^31–33^.

Here, we applied atomistic discrete molecular dynamics (DMD), a predictive and computationally efficient molecular dynamics approach^34–36^, to investigate the assembly dynamics^37^ of the aforementioned seven peptides. β-barrel oligomers were observed for the cytotoxic hIAPP19–29 and its S20G mutant as well as hIAPP22–28, Aβ16–22 and NACore. The β-barrels, corresponding to “closed” β-sheets mainly formed by six to eight peptides, were the aggregation intermediates that converted into multi-layer β-sheets with increasing oligomer sizes. The inter-conversion between closed β-barrels and open β-sheets with single or double layers was observed in DMD simulations. For these five peptides, the final aggregates in simulations of large molecular systems resembled the cross-β protofibrils consistent with the experimentally-observed mated β-sheets. Nontoxic hIAPP15–25 and hIAPP(S20G)15–25, on the other hand, first assembled into mostly unstructured and loosely compact oligomers, in which the β-sheet contents gradually increased with increasing oligomer sizes. The β-sheet rich aggregates of hIAPP15–25 and its S20G mutation were polymorphic without forming the mated multi-layer β-sheets^27^. While previous studies attributed the differential toxicity between hIAPP19–29 and hIAPP15–25 to their different aggregation morphologies^27^, our results suggest that the toxicity might be mediated by the formation of β-barrel oligomers although the question of how these oligomers cause cytotoxicity remains to be uncovered with future experimental and computational studies. Hence, we postulate that β-barrel oligomers are common aggregation intermediates towards the final formation of cross-β aggregates and these β-barrel oligomer intermediates, among many other factors^38,39^, may contribute to the cytotoxicity of amyloid aggregation.

## Result and Discussion

We first focused on the oligomerization and fibrillization dynamics of hIAPP15–25, hIAPP19–29, and their S20G mutants. For each of the four sequences including hIAPP15–25, hIAPP(S20G)15–25, hIAPP19–29 and hIAPP(S20G)19–29, ten molecular systems with even number of peptides from 2 to 20 were studied (Methods). In all cases, the same peptide concentration was maintained by adjusting the simulation box size. For each molecular system, ten independent DMD simulations lasted 300 ns at 300 K were performed starting with different initial coordinates (e.g., different inter-molecular distances and orientations) and velocities. The equilibration of each peptide system in simulations was first assessed according to the time evolution of secondary structure properties (e.g., the main conformation states of random coil and β-sheet contents) and energetics (e.g., potential energy and the number of backbone hydrogen bonds), which reached their steady states after 150 ns in all simulations (e.g., representative trajectories of the largest molecular systems of 20 peptides for each of four sequences in Fig. S1).

### IAPP19–29 had a higher β-sheet propensity than hIAPP15–25 during aggregation

**Figure 1:**
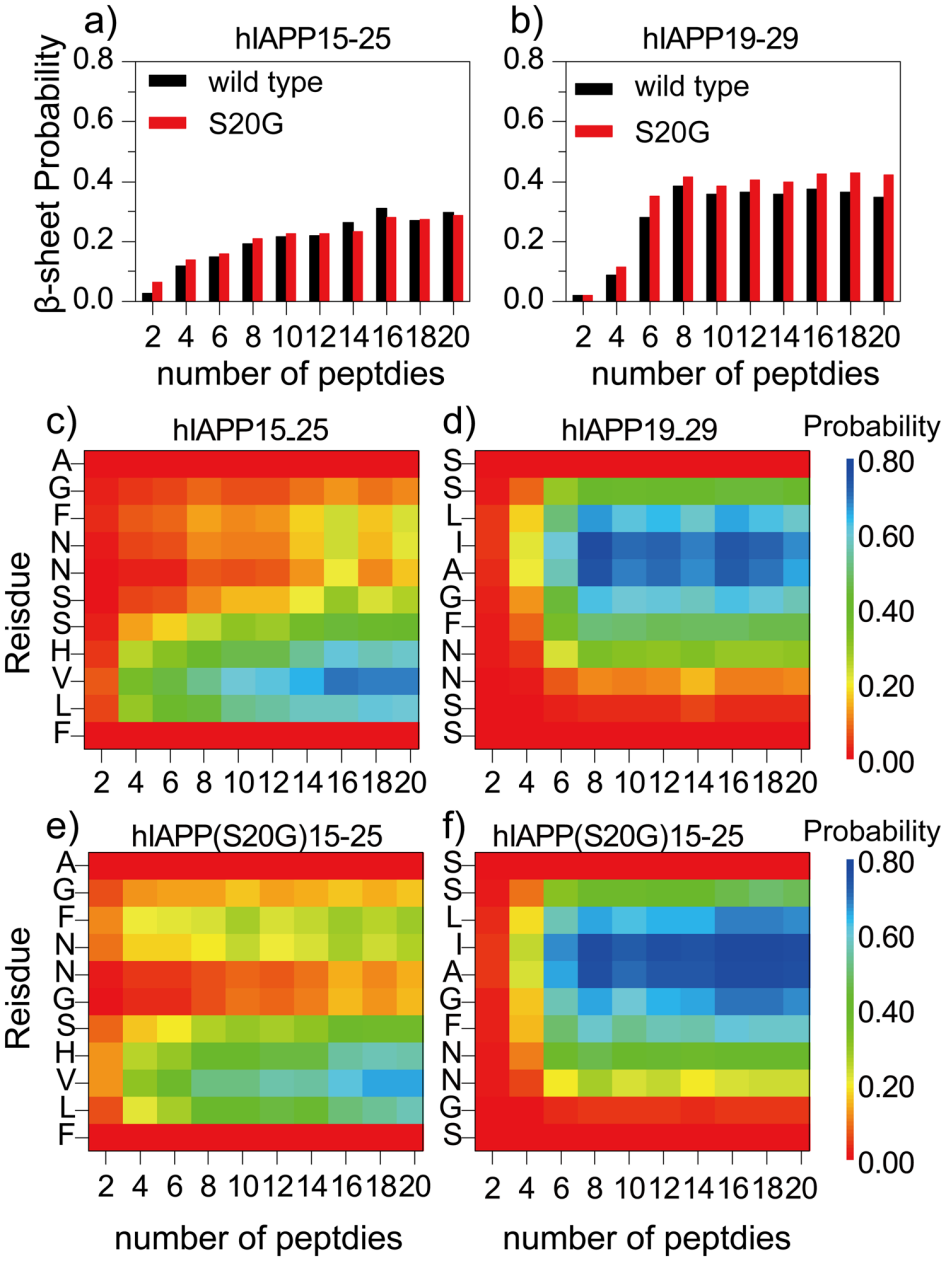
The averaged β-sheet content (**a**,**b**) and the β-sheet propensity per residue (**cf**) in simulations with increasing number of peptides for each of the four sequences of hIAPP15–25, hIAPP(S20G)15–25, hIAPP19–29, and hIAPP(S20G)19–29. For each molecular system, the last 150 ns simulations of ten independent runs were used for secondary structure analysis.

We first examined the peptide secondary structure properties with increasing system sizes (β-sheet in Fig. 1 and random coil in Fig. S2). The second half of each trajectory was used for the calculation of equilibrium properties as suggested by the equilibration analysis (Fig. S1). For both hIAPP15–25 and S20G mutant, the content of β-sheet increased and random coil decreased with increasing number of peptides in simulations (Fig. 1a). Examination of the β–sheet probability per residue (Fig. 1c,e) suggested that two separate regions near the N- (e.g., L16, V17, H18, and S19) and C-terminus (e.g., N22 and F23) had high β–sheet propensities. While the S20G mutation in the middle of the sequence of hIAPP15–25 did not affect the overall β-sheet contents, it slightly increased the β–sheet propensity of the C-terminal region and weakened the N-terminal region. The secondary structure contents of hIAPP19–29 and hIAPP(S20G)19–29_,_ on the other hand, exhibited a sharp coil-to-sheet transition with respect to the simulation system size (Fig. 1b). With less than six peptides, the peptides showed a weak β-sheet propensity, but as the number of peptides increased to six and larger the β–sheet content was significantly enhanced. Except residues near the termini, all other residues around the amyloidogenic core sequence of full-length hIAPP (^22^NFGAIL^27^) had a high propensity to form β–sheet (Fig. 1d,f). The S20G mutation at the second residue of hIAPP19–29 promoted the overall β-sheet content by also increasing the β–sheet propensity of residues following it in sequences.

### hIAPP15–25 formed bent parallel β–sheets while hIAPP19–29 aggregated into extended β-sheets with mixed parallel and anti-parallel alignments

**Figure 2:**
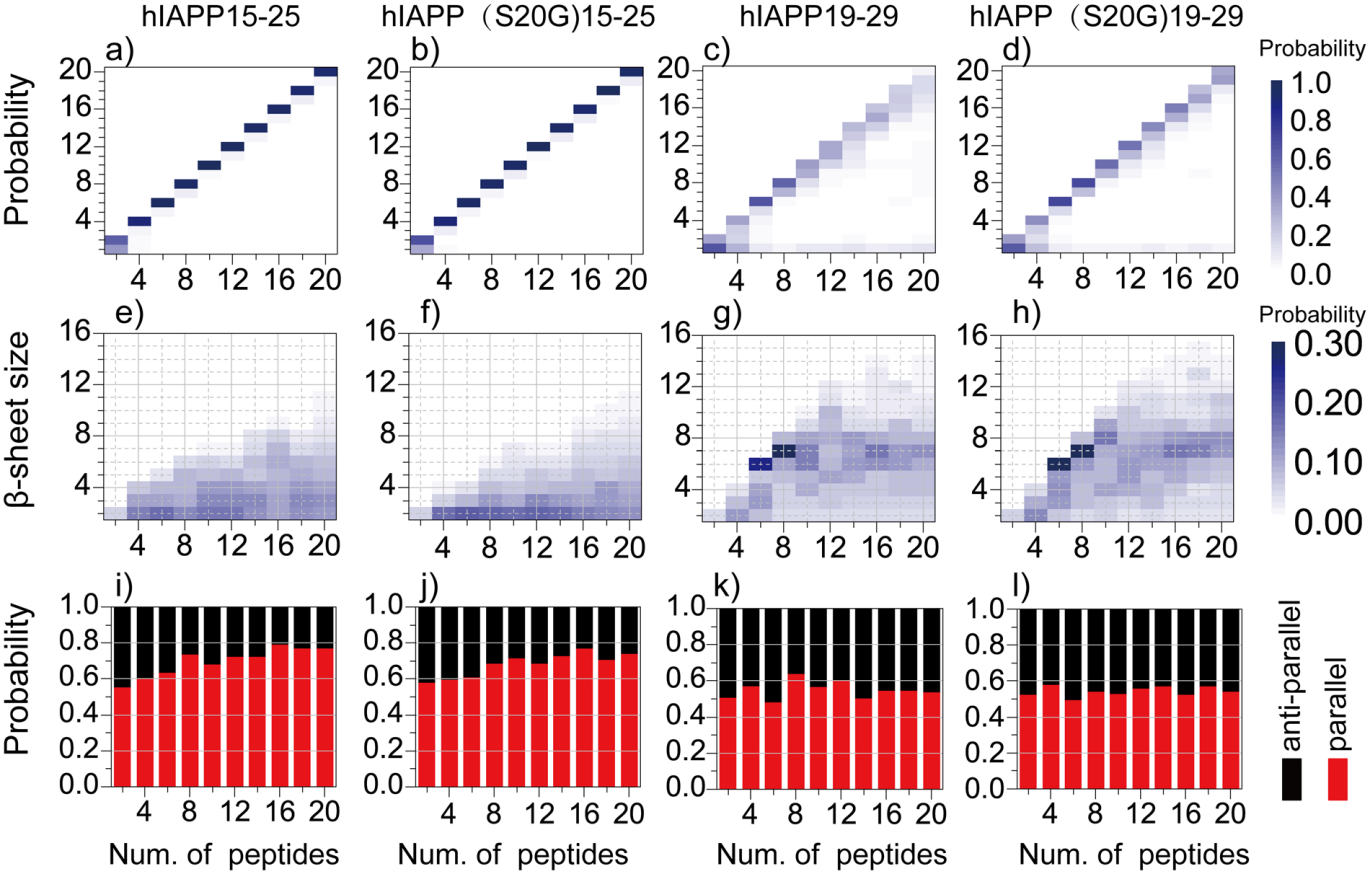
Structural analysis of oligomers and β-sheets. The changes in the probability distributions of (**a**–**d**) oligomer sizes and (**e**–**h**) β-sheet sizes with increasing number of peptides in DMD simulations of hIAPP15–25, hIAPP(S20G)15–25, hIAPP19–29, and hIAPP(S20G)19–29. The probabilities were color coded according to the color bars. (**i**–**l**) The percentages of anti-parallel (black bar) and parallel (red bar) alignments between neighboring β-strands was also shown for different sequences in simulations with different number of peptides.

We studied the peptide assembly dynamics by monitoring the oligomer formation and characterizing the structures of β–sheets in these aggregates. An oligomer was defined as a cluster of peptides connected by inter-molecular heavy atom contacts and its size was defined as the number of peptides forming the oligomer. By averaging over trajectories of independent simulations, we computed the mass-weighted oligomer size distribution (Fig. 2a–d), corresponding to the probability of finding a peptide in a given size oligomer. hIAPP15–25 and hIAPP(S20G)15–25 tended to self-associate into a single oligomer with the oligomer size equal to the total number of peptides in simulations (Fig. 2a,b). The oligomerization process of hIAPP19–29 and hIAPP(S20G)19–29 was more dynamic with significant populations of many smaller oligomers and even monomers (Fig. 2c,d). The higher self-association/oligomerization propensity of hIAPP15–25 was due to its higher overall sequence hydrophobicity than hIAPP19–29. Excluding the overlapping region, the sequence from 15–18 (FLVH) is more hydrophobic than residues 26–29 (ILSS).

To characterize β–sheet structures, we computed the mass-weighted size distribution of β-sheets, whose size corresponded to the number of β-strands forming the sheet (Fig. 2e–h). hIAPP15–25 and hIAPP(S20G)15–25 tend to form small β-sheets with the effect of mutation reducing the sizes. hIAPP19–29 and hIAPP(S20G)19–29, on the other hand, preferred to form larger β-sheets when the number of peptides was six and bigger after the coil-to-sheet transition (Fig. 1b). For example, the most populated β-sheet sizes were six and seven for simulations of six and eight peptides, respectively, suggesting that the peptides preferred to form a single-layer β-sheet. As the system size increased to ten or more, the most dominant β-sheet layer size kept around 6~8, indicating that the peptides might form multi-layer β-sheets. The analysis of the alignments between neighboring β-strands in each β-sheet indicated that hIAPP15–25 and hIAPP(S20G)15–25 displayed a high propensity (~0.6–0.8) to form parallel β-sheets, and the β-sheets of hIAPP19–29 and hIAPP(S20G)19–29 aggregates had both parallel and anti-parallel alignments of β-strands with a ratio ~1:1 (Fig. 2i–l). We also calculated the probability distribution of β-strand length (defined as the number of consecutive residues in a peptide adopting β-sheet conformation in Fig. S3) and the end-to-end distance of each peptide (Fig. S4). hIAPP15–25 mainly formed short β-strands (~2–5 residues), and the longer β-strands (~7–9 residues) were also observed with smaller probabilities when the number of peptides was larger than six (Fig. S3a). The mutation in hIAPP(S20G)15–25 rendered the β-strands shorter with the probability to form longer β-strands decreased (Fig. S3c). Different from hIAPP15–25, the β-strands of 5–8 residues was the most populated conformation for both hIAPP19–29 and hIAPP(S20G)19–29 and the mutant had β-strands of 6–8 residues more populated (Fig. S3b,d). The end-to-end distances of hIAPP19–29 and hIAPP(S20G)19–29 were larger than that of hIAPP15–25 and hIAPP(S20G)15–25 (Fig. S4). The S20G mutation induced hIAPP15–25 less extended, but more extended for hIAPP19–29.

### hIAPP19–29 and its S20G mutant formed β-barrel oligomers as the aggregation intermediates

**Figure 3:**
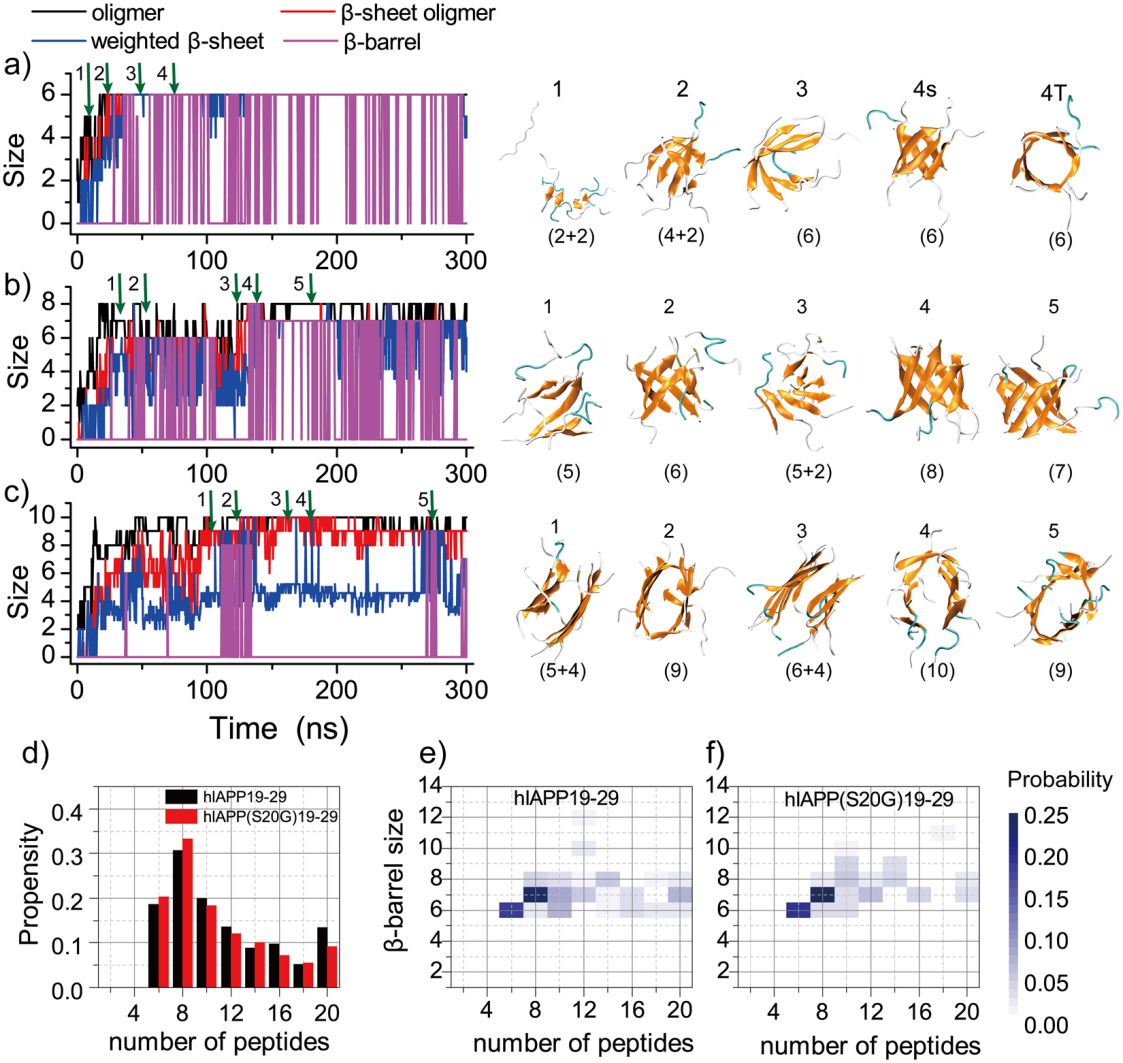
The aggregation dynamics of hIAPP19–29. The self-assembly dynamic of (**a**) six, (**b**) eight and (**c**) ten hIAPP19–29 peptides were monitored by the time evolution of the largest oligomer size (black), the largest β-sheet oligomer size (red), the mass-weight average β-sheet size (blue) and the total β-barrel size (purple) in each representative trajectory. The snapshot structures along the simulation trajectories as indicated by green arrows were presented on the right lane. Each peptide was shown in cartoon representation with strand colored in yellow, coil in gray, and turn in cyan. The sizes of β-sheets were given in the parentheses. (**d**) The average probability of a peptide to form β-barrel oligomers was computed for hIAPP19–29 and hIAPP(S20G)19–29 in simulations of different number of peptides. (**e**,**f**) The changes in the probability distributions of β-barrel sizes with increasing number of peptides in DMD simulations of hIAPP19–29 and hIAPP(S20G)19–29.

Ensemble average analysis suggested that the oligomers of hIAPP19–29 formed single- or multi-layer β-sheets when the number of peptides was six or bigger after the coil-to-sheet transition. We further investigated the conformational dynamics of these oligomers along the simulation trajectories, and found that these β-sheets could adopt closed forms as β-barrels (Fig. 3). For each snapshot along a trajectory, we monitored the size of the largest oligomer and the largest β-sheet oligomer, the mass-weighted average size of β-sheets, and the total size of the β-barrels (details in Methods). The β-barrels were observed in simulations of at least six peptides. In simulations of six peptides (e.g., a typical trajectory in Fig. 3a), hIAPP19–29 rapidly assembled into oligomers with multiple small β-sheets with short β-strands (e.g., snapshots 1 and 2 in Fig. 3a), where these small β-sheets grew into a single sheet with longer β-strands (e.g., snapshot 3 in Fig. 3a). The single-layer β-sheet could rearrange into a hexameric β-barrel (e.g., snapshot 4 in Fig. 3a), which underwent an open-and-close dynamics during the course of simulations (i.e., the fluctuations of β-barrels after 50 ns without changes in β-sheet sizes in Fig. 3a). These β-barrels structurally resemble the experimentally-determined cylindrin aggregates of the αB crystallin fragment^11^ and also computationally-observed β-barrels of Aβ16–22^20,23,40^ and the fragment of beta-2 microglobulin (β2m83–89)^21^. A similar self-assembly dynamics was observed for simulations of eight hIAPP19–29 peptides (Fig. 3b), where hexamer, heptamer and octamer β-barrels were observed (e.g., snapshots 2, 5 and 4 in Fig. 3c, respectively). However, when the number of peptides increased to ten in simulations (Fig. 3c) we found that hIAPP19–29 predominantly formed two-layer β-sheets with the size of the largest β-sheet oligomers (red line) twice of the average size of β-sheet (blue line) although β-barrels were still transiently observed. The conformational inter-conversions of β-barrels with single-layer (e.g., snapshots 2 and 5 in Fig. 3c) and two-layer (e.g., snapshots 1 and 3 in Fig. 3c) β-sheets were observed, suggesting comparable free energies among these aggregations intermediate species. This conformational inter-conversion was also detected in prior computational aggregation studies of amyloid fragments (e.g., Aβ16–22, β2m83–89) using both all-atom and coarse-grained simulations^20,21,23,40,41^. The mutant hIAPP(S20G)19–29 featured the same oligomerization dynamics as hIAPP19–29 (Fig. S5).

We further computed the probability of a peptide forming β-barrels during last 150 ns simulations (Fig. 3d). Both hIAPP19–29 and hIAPP(S20G)19–29 peptides showed a high β-barrel propensity in simulations of six, eight and ten peptides with a probability around 20%, 30% and 20%, respectively. As the number of peptides increased, the β-barrel probability gradually decreased to ~10% due to the increased preference to form multi-layer β-sheets as observed in simulations of ten peptides (Fig. 3c). We also computed the probability distribution of β-barrel sizes (Fig. 3e,f), where the most populated β-barrels were composed of ~6–8 β-strands with larger β-barrels being observed occasionally. The observed β-barrel oligomer sizes also agree with another recent experimental study, where Aβ25-35 formed the most efficient β-rich pores with the number of peptides ranging from 6 to 8^42^. Therefore, our simulation results revealed the propensity of hIAPP19–29 and its S20G mutant to form β-barrel oligomers as the aggregation intermediates.

**Figure 4:**
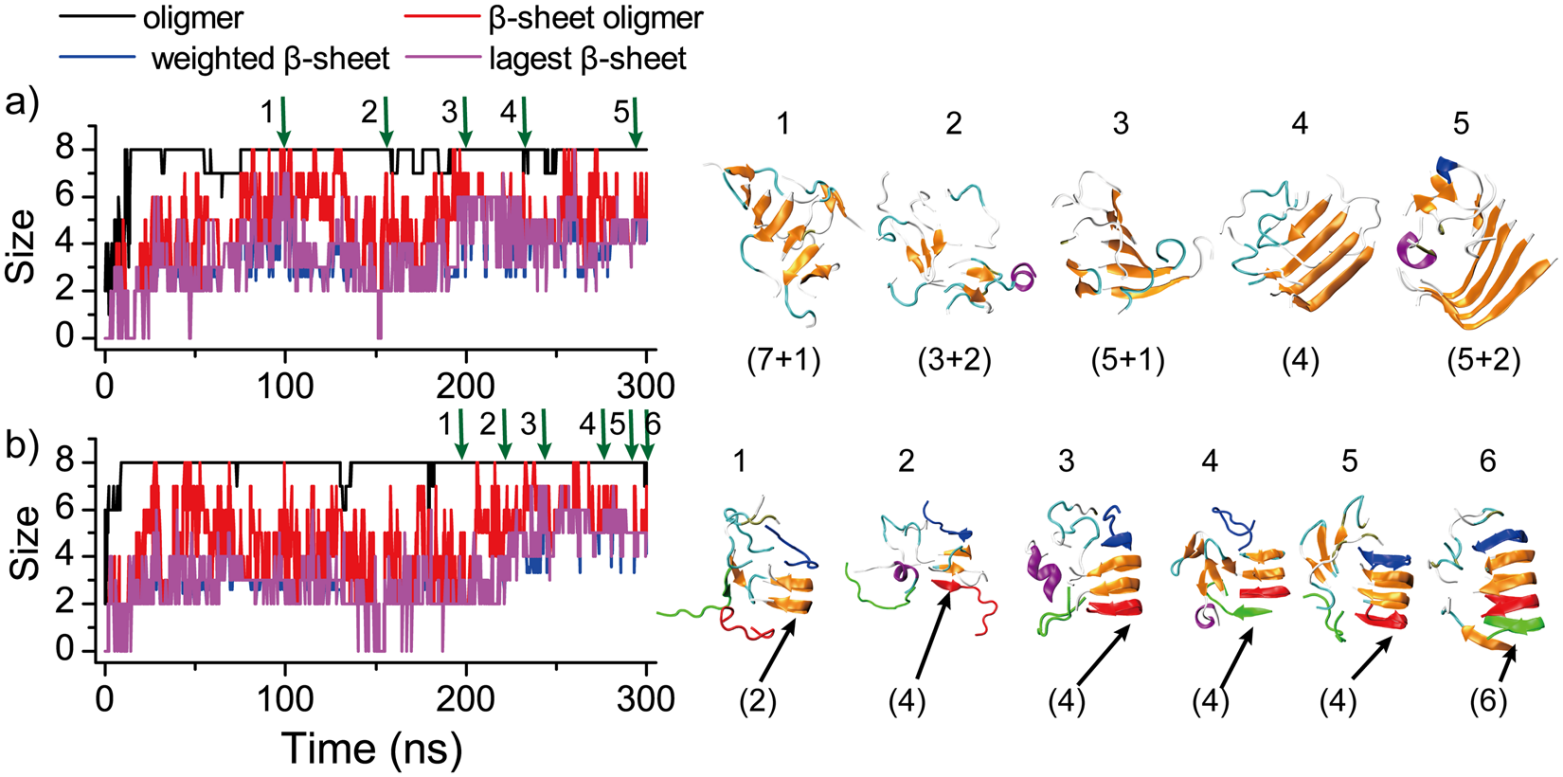
The aggregation dynamics of hIAPP15–25. The aggregation processes of eight peptides from isolated random coil conformations to single-layer β-sheets with either L-turn (**a**) and U-turn (**b**) morphologies. The largest oligomer size (black), the largest β-sheet oligomer size (red), the mass-weighted average β-sheet size (blue) and the largest β-sheet size (purple) were plotted as the function of simulation time. The snapshot structures along the simulation trajectories as pointed by green arrows were given on the right lane, where each peptide was shown in cartoon representation with strand colored in yellow, coil in gray, helix in purple, and turn in cyan. The sizes of β-sheets were given in the parentheses. In panel b, three initially unstructured peptides (colored in red, blue and green, respectively) were highlighted to illustrate their binding, structural rearrangement, and eventually incorporation into the final β-sheets.

hIAPP15–25 and the S20G mutant formed polymorphic β-sheet aggregates. We applied the same conformational dynamics analysis for the aggregation of hIAPP15–25 (Fig. 4) and hIAPP(S20G)15–25 (Fig. S6). No stable β-sheet oligomers were observed when the number of peptides less than eight for hIAPP15–25 (Fig. 2e) and ten for hIAPP(S20G)15–25 (Fig. 2f). As the number of hIAPP15–25 peptides increased to eight or more in aggregation simulations, we found that the peptides could form two types of parallel β-sheets, one bent near the C-terminal (L-turn, Fig. 4a) and the other bent in both N- and C-termini (U-turn, Fig. 4b), consistent with a wide distribution of end-2-end distances (Fig. S4a). Using the network-based algorithm of detecting β-barrel formations^40^, we scanned all independent simulations for all simulated molecular systems and the β-barrel oligomers were not observed in any of the simulations for the two sequences. Since the steady states were achieved in all simulations (e.g., Figs 4, S1, S6 and S7), we do not expect to observe β-barrel conformations of hIAPP15–25 and its S20G mutant with longer simulations.

As shown in Fig. 4a of a typical aggregation trajectory with eight peptides, hIAPP15–25 first collapsed into a single coil-rich oligomer (within the first 10 ns), where the peptides started to form β-sheets with short β-strands (e.g., snapshot 1 in Fig. 4a). These β-sheets were unstable and underwent frequent conformational rearrangement (e.g., snapshots 2–4 in Fig. 4a), finally forming a stable L-turn β-sheet (e.g., snapshot 5 in Fig. 4a). The peptide could also follow similar aggregation dynamics to form the U-turn β-sheet aggregates (Fig. 4b). The sequence asymmetry in terms of hydrophobicity and β-sheet propensity (i.e., the N-terminal of hIAPP15–25 was more hydrophobic with a higher β-sheet propensity compared to the C-terminal as shown in Fig. 1c,e) drove the predominantly parallel alignments of stable β-sheets. For example, due to the high hydrophobicity, the N-terminal of an hIAPP15–25 could bind to the sides of a performed β-sheet at N-terminals first via either parallel or anti-parallel alignments (e.g., snapshots 1–3 in Fig. 4b). Since the anti-parallel alignment between the N-terminals of different chains were less stable compared to the parallel alignment which was stabilized by additional hydrophobic interaction between the C-terminals, a peptide bound with the initially anti-parallel alignment could easily dissociate and re-associate to form a more stable parallel β-sheet (e.g., snapshots 4–6 in Fig. 4b). The U-shaped β-sheet structures of hIAPP15–25 were consistent with the same sequence fragments in the fibril models of full length hIAPP reconstructed by either solid-state NMR constraints^43^ or X-ray crystallography studies of fibril structures of composite peptides^44^. A recent NMR study of hIAPP binding with an aggregation inhibitor also featured a β-hairpin conformation of hIAPP around residues 20–21^45^. For the mutant hIAPP(S20G)15–25, we didn’t observe any stable β-sheet oligomers in simulations up to eight peptides, but L-turn and U-turn β-sheet oligomers with similar aggregation dynamics were also observed in simulations of ten (Fig. S6) or more peptides. Therefore, both hIAPP15–25 and hIAPP(S20G)15–25 formed polymorphic β-sheet aggregates without β-barrel oligomers as the aggregation intermediates.

### The aggregation free energy landscape in terms of oligomerization and fibrillization

**Figure 5:**
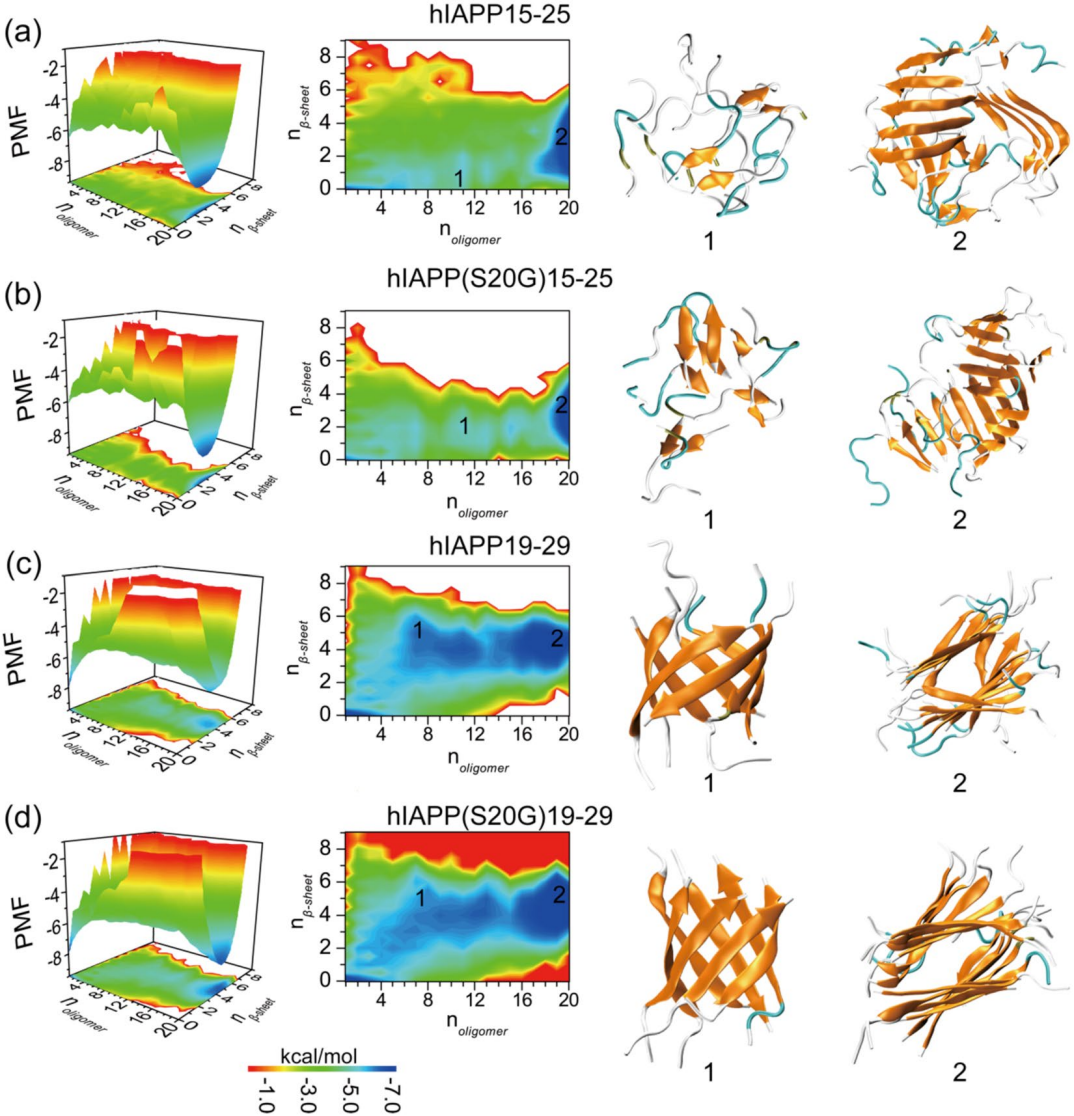
The aggregation free energy landscape. The PMF (i.e., the effective free energy) as a function of the oligomer size *n*_*oligomer*_ and the average number of residues adopting β-sheet structure per chain, *n*_*β-sheet*_, in the aggregation of 20 peptides were shown for each of the four sequences including (**a**) hIAPP15–25, (**b**) hIAPP(S20G)15–25, (c) hIAPP19–29, and (d) hIAPP(S20G)19–29. To capture the initial aggregation dynamics, the analysis included the whole 300 ns trajectories of 10 independent runs. Both three-dimensional (left) and two-dimensional (middle) representation of the PMF were shown. Snapshot structures of the intermediates and final aggregates labeled as 1 and 2 in the PMF plot were also shown to the right. Each peptide was shown in cartoon representation with strand colored in yellow, coil in gray, and turn in cyan.

To better understand the aggregation process, we computed the potential of mean force (PMF, i.e., the effective free energy), widely used in studying amyloid aggregation kinetics^37,46^, as a function of the oligomer size (*n*_*oligomer*_) and the number of residues in β-sheet structure per peptide (*n*_*β-sheet*_) for simulations with 20 peptides (Fig. 5a–d). All the 300 ns trajectories from 10 independent simulations were included in the analysis to capture the early assembly process. The aggregation free energy landscape of hIAPP15–25 featured two well-defined basins around (1, 0) and (20, 3), corresponding to isolated monomers at the initial stage of aggregation and the final β-sheet rich aggregates (e.g., Fig. 5a). Oligomers less than 20 peptides showed weak β-sheet contents. Examination of the assembly dynamics (e.g., a representative trajectory in Fig. S7a,e) showed that driven by hydrophobic interactions hIAPP15–25 first rapidly associated into a single oligomer without forming extensive hydrogen bonds (within ~50 ns, Fig. S7a), where more β-sheets were gradually formed with increasing number of inter-chain hydrogen bonds (e.g., 50–150 ns). During the structural rearrangement within the large oligomer, the number of backbone hydrogen bonds increased mainly between parallel β-sheet (Fig. S7e), resulting into the predominantly parallel β-sheets in the aggregates (Fig. 2). The inter-peptide contact frequency map between backbones of different residues (Fig. S8a) confirmed the in-register parallel β-sheets, especially in the N-terminal. The side-chain contract frequency map (Fig. S8b) also revealed the strong hydrophobic interaction among N-terminal residues and their interaction with the C-terminal ^23^F as in the U-turn β-sheets (e.g., Fig. 4b). In the final aggregates of hIAPP15–25 (e.g., Fig. 5a), different β-sheets with bent conformations were not aligned with each other, in agreement with the experimentally-observed labile and unmated β-sheets formed by the same sequence^27^. The hIAPP(S20G)15–25 mutant showed a similar aggregation free energy landscape and aggregation dynamics as hIAPP15–25 (Figs 5b, S7 and S8).

There were also two deep free energy basins for the aggregation of hIAPP19–29 around (1, 0) and (20, 5) corresponding to initial monomers and final β-sheet rich aggregates, but the aggregation pathways and dynamics were drastically different from hIAPP15–25 (Fig. 5c). The aggregation of hIAPP19–29 featured smaller oligomers with high β-sheet contents *en route* to the final aggregates (e.g., Fig. S7c). Smaller oligomers with less than six peptides had little β-sheet content, and β-sheet rich oligomers started to form with six or more peptides (Fig. 5c). Analysis of the assembly dynamics (e.g., a representative trajectory in Fig. S7c,g) confirmed the initial formation of small β-sheet rich oligomers and the growth of large β-sheet oligomers via either the self-association of small oligomers (e.g., the large step-wise increase of oligomer sizes around 75 ns in Fig. S7c) or the addition of monomers (e.g., the small step-wise fluctuations in oligomer sizes in Fig. S7c). The β-barrel intermediates were frequently observed during the aggregation process (e.g., snapshot in Fig. 5c and purple lines in Fig. S7c). The backbone contact frequency maps also revealed that both parallel in-register and anti-parallel out-register β-sheets were formed during aggregation (Fig. S8a). The final aggregates (e.g., Fig. 5) were comprised of two β-sheets face-to-face with side-chains inter-digitation of central residues (e.g., the side-chain contact frequency map in Fig. S8b), also consistent with the experimentally determined fibril structures of the same sequence^27^. hIAPP(S20G)19–29 showed a similar aggregation behavior as hIAPP19–29, except with the basin of the final aggregates having lower free energies (Figs 5d, S7d,h and S8).

### Other amyloid peptides, including hIAPP22–28, Aβ16–22 and NACore, could also form **β-barrel** oligomer intermediates

**Figure 6:**
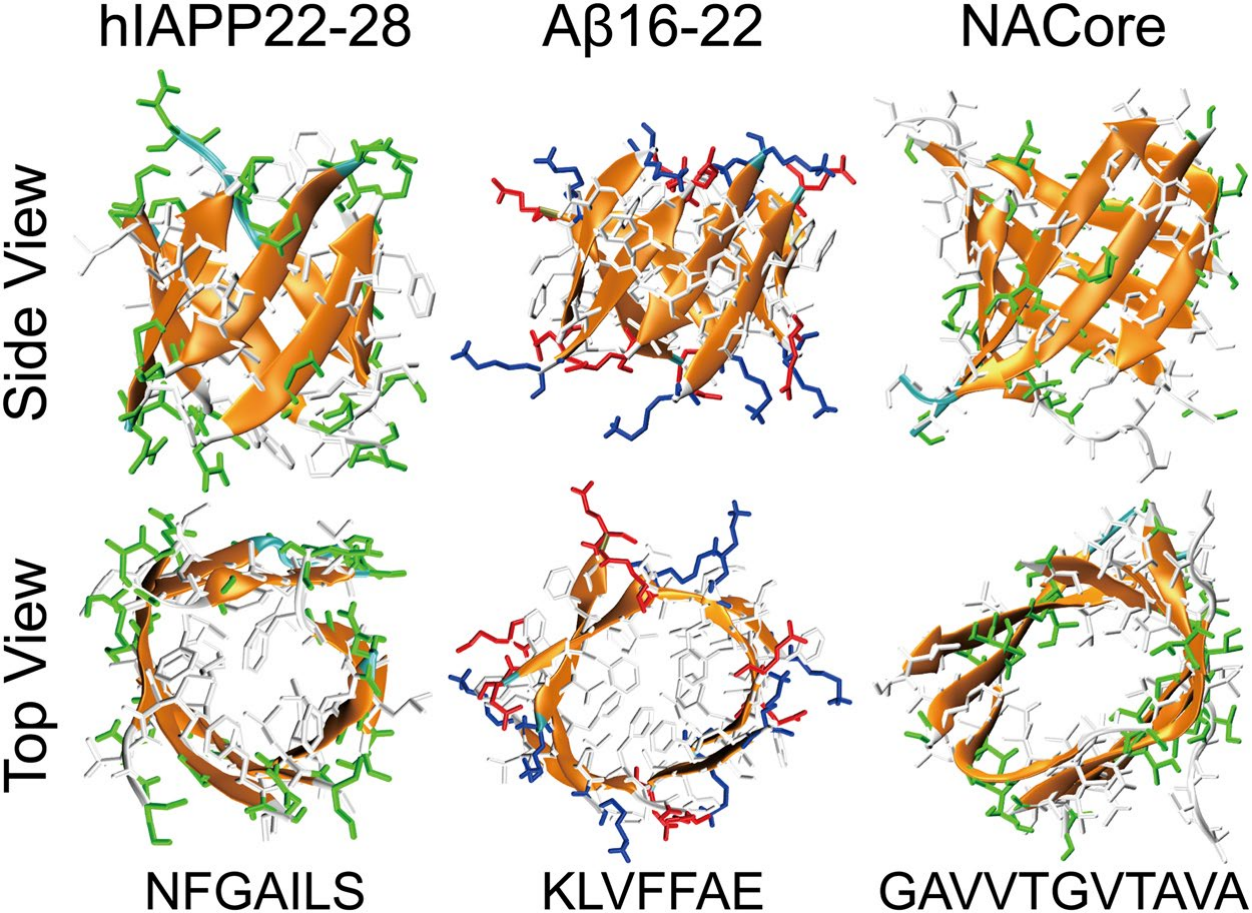
Typical β-barrel oligomers formed by hIAPP22–28, Aβ16–22 and NACore shown with both top and side views. The peptides were shown in cartoon representation with side-chains shown as sticks and colored according to residue types (hydrophobic in white, hydrophilic in green, positively charged in blue, and negatively charged in red). The sequence of each amyloid fragment was also given at the bottom.

It is widely accepted that the toxicity of amyloid proteins shared a similar mechanism. For example, the toxicity of amyloid peptides, including amyloid-β, α-synuclein and serum amyloid A, hIAPP, was linked to membrane damage by the formation of amyloid channels (i.e., “amyloid pores”) in the membrane^47,48^. To investigate whether the β-barrel oligomers are also formed in the aggregation of other amyloid peptides, we analyzed the aggregation dynamics of three other amyloid peptides, including hIAPP22–28^31^, Aβ16–22^32^ and NACore^33^. For each sequence, ten independent aggregation simulations of eight peptides were performed starting with fully extended conformations and random positions and inter-peptide orientations (Methods). Indeed, all three peptides could aggregate into well-organized β-barrel structures, with hydrophobic residues buried and polar residues solvent-exposed (Fig. 6). The probability of observing β-barrel intermediates for hIAPP22–28, Aβ16–22 and NACore was ~1.2%, 7.1% and 1.9%, respectively.

In our recent work on the differential aggregation pathways between hIAPP22–28 and Aβ16–22 peptides, we found that with up to 16 peptides in aggregation simulations the final aggregates of both peptides adopted cross-β structures^49^. The final aggregates of ten NACore peptides also adopted two-layered cross-β structure (Fig. S9), which was consistent with X-ray diffraction studies^33^. Using all-atom REMD simulations with explicit solvent, Aβ16–22^20^ and an amyloidogenic segment of SOD1 (residues 147–153)^50–52^ were found to be able to form both β-barrel oligomers and two-layer β-sheets (i.e., the cross-β like aggregates). The β-barrel oligomers observed in this work and prior computational^20,40,50–52^ or experimental^11^ studies were composed of single-layer β-sheets, different from the double-layer β-barrel model proposed to constitute the amyloid channel across a cell membrane^53–55^. It remains to be uncovered whether full-length amyloid peptides or larger number of peptide fragments could spontaneously form the postulated double-layer β-barrels in solution or the membrane environment. Taken together, all of these data suggest that β-barrel oligomers are common intermediates towards the formation of cross-β fibrils in amyloid aggregation.

During the early aggregation stage of cross-β-forming peptides when β-sheets are initially nucleated, small two-layer β-sheets can be formed readily. These β-sheets prefer to associate with each other via parallel or anti-parallel alignments of their composite β-strands as in the final aggregates. With relatively low thermal stability and thus large conformational flexibility, peptides at the ends of these two-layer β-sheets can join each other by form hydrogen bonds along the backbone and thus these two-layer β-sheets convert into either “open” or “closed” single β-sheets, the latter of which correspond to β-barrels. Hence, this aggregation scenario suggests the co-existence of β-barrels, curved single β-sheets, and two-layer β-sheets during the early aggregation stage before the final formation of cross-β fibrils (e.g., as illustrated during the aggregation dynamics in Fig. 3 and aggregation free energy landscape in Fig. 5 of hIAPP19–29).

## Conclusion

In summary, we computationally investigated the aggregation dynamics of several well-studied peptides derived from various amyloidogenic proteins. Consistent with experimentally observed morphologies of final aggregates^27^, hIAPP19–29 and its S20G mutant tended to form two-layer β-sheets face-to-face with hydrophobic side-chains packed against each other in our simulations; but the β-sheets of hIAPP15–25 and the S20G mutant were polymorphic with different bent conformations that did not form the mated β-sheet packing. Similarly, hIAPP22–28, Aβ16–22 and NACore all formed the cross-β aggregates in agreement with experiments^31–33^ or molecular dynamics simulations with explicit solvent^20^. Hence, the ability to recapitulate aggregate morphologies of all seven peptides underscores the predictive power of our all-atom DMD with implicit solvent.

In addition to the aggregate morphologies, we also analyzed the oligomerization dynamics and evaluated the formation of β-barrel oligomer intermediates. We found that β-barrel oligomers were common intermediates for peptides assembling into the cross-β like aggregates, including hIAPP19–29, hIAPP(S20G)19–29, hIAPP22–28, Aβ16–22 and NACore. For example, oligomers of hIAPP19–29 featured a coil-to-sheet conformational transition after their sizes increased to six or larger. The β-barrel oligomers mainly comprised of ~six-eight β-strands were observed as the aggregation intermediates and structurally inter-converted with single- and double-layer β-sheets. hIAPP15–25 and the S20G mutant, on the other hand, did not β-barrel oligomer. Instead, the peptides tended to associate with each other into large coil-rich oligomers, within which β-sheets were gradually formed. Together with previous computational studies of individual sequences, our results suggest β-barrel oligomers might be the common aggregation intermediates of peptides that assembly into cross-β amyloid aggregates.

Without forming β-barrel oligomers as aggregation intermediates, the hIAPP15–25 and hIAPP(S20G)15–25 were nontoxic *in vitro*^27^. On the other hand, the other peptides that had β-barrel oligomer intermediates during aggregation were all documented to be toxic in the literature. The correlation between the formation of β-barrel oligomer intermediates and cytotoxicity supports to the hypothesis of β-barrel oligomers as the toxic oligomers in amyloid aggregation^11^. While Krotee *et al*.^27^ attributed the differential toxicity between hIAPP15–25 and hIAPP19–29 to the different morphology of β-sheet aggregates, our results suggest that the observed toxicity might be mediated by β-barrel oligomers formed by hIAPP19–29 instead of hIAPP15–25.

β-barrel oligomers observed here and in previous experimental^11^ and computational computational^20,40,50–52^ studies were formed by peptide fragments derived from amyloid proteins. Although there is no direct structural evidence for beta-barrel oligomers of full-length amyloid proteins, indirect experimental evidence based on hydrogen exchange mass spectrometry^24^ and NMR^25^ supports the formation of β-barrel oligomers by Aβ40 and Aβ42. Future studies are required to uncover the structure and dynamics of β-barrel oligomers formed by full-length amyloid proteins. In addition, to understand how the β-barrels interact with membrane and cause membrane damage, it is also necessary to uncover the aggregation of amyloid peptides in the membrane environment and to study the formation of the membrane-associated β-barrels.

## Materials and Methods

### Molecular systems used in simulations

We systematically investigated the assembly dynamics of hIAPP15–25 and hIAPP19–29 and their S20G mutants (denote as hIAPP(S20G)15–25 and hIAPP(S20G)19–29). To capture the self-assemble dynamics and oligomer structure at different size of these four types peptide, 10 systems were setup with even number of peptides from 2 to 20 for each fragment, each system performed 300 ns ten independently DMD simulation with different initial configurations (i.e., coordinates and velocities). For hIAPP22–28, Aβ16–22 and NACore, only aggregation simulations with eight peptides were performed. For each of the three cytotoxic peptides, ten independent DMD simulations with each trajectory lasting 200 ns were carried out. In all cases, the same peptide concentration of ~26 mM was maintained by adjusting the simulation box sizes. The details of all the simulations were summarized in Table 1.

**Table 1:** The details of molecule systems in our DMD simulations, including the number of peptides (*N*_*peptide*_), the corresponding dimension of the cubic simulation box, the number of DMD runs (*N*_*run*_), the length of each DMD simulations, and the accumulative total simulation times.

hIAPP15–25, hIAPP19–29 and S20G mutants										
*N*_*peptide*_	2	4	6	8	10	12	14	16	18	20
Dimension, nm	6.1	6.3	7.2	7.9	8.5	9.0	9.6	10	10.4	10.8
*N*_*run*_	10	10	10	10	10	10	10	10	10	10
Time, ns	300	300	300	300	300	300	300	300	300	300
Total time, µs	3.0	3.0	3.0	3.0	3.0	3.0	3.0	3.0	3.0	3.0
**hIAPP22–28, Aβ16–22, NACore**										
*N*_*peptide*_	2	4	6	8	10	12	14	16	18	20
Dimension, nm	—	—	—	7.9	—	—	—	—	—	—
*N*_*run*_	—	—	—	10	—	—	—	—	—	—
Time, ns	—	—	—	200	—	—	—	—	—	—
Total time, µs	—	—	—	2.0	—	—	—	—	—	—

### Details of DMD simulations

All simulations were performed in canonical (NVT) ensemble using the discrete molecular dynamics^56,57^ (DMD) algorithm. DMD is a unique type of molecular dynamics algorithm with significantly enhanced sampling efficiency, which has been wildly used by our group and other in studying protein folding^36^ and amyloid peptides aggregation^58^. In DMD simulations, the inter-atomic interactions were modeled by discrete step-wise functions mimicking the continuous potential functions of the conventional molecular mechanics force fields. Bonded interactions (bonds, bond angles, and dihedrals) were modeled as infinite square wells, where covalent bonds and bond angles usually have a single well and dihedrals may feature multiple wells corresponding to cis- or trans-conformations. Non-bonded interactions (i.e., van der Waals, solvation, hydrogen bond, and electrostatic terms) were represented as a series of discrete energetic steps, decreasing in magnitude with increasing distance until reaching zero at the cutoff distance. The van der Waals parameters were adopted from the CHARMM force field^59^, and bonded termed were parameterized based on statistical analysis of protein structures from protein data bank (PDB). The water molecules were implicitly modeled using the EEF1 implicit solvation model developed by Lazaridis and Karplus^60^. A reaction-like algorithm was used to model hydrogen bonds^61^. The electrostatic interactions were screened using the Debye-Hückel approximation with screening length set to 10 Å, which corresponds to ~100 mM of *NaCl* under physiological conditions. The velocity of each atom kept constant unless a collision occurred when an inter-atom potential was change, then the velocity was updated following the conservation laws of energy, momentum and angular momentum. The units of time, length, and energy were ~50 femtosecond, 1 Å, and 1 kcal/mol, respectively. The temperature of the system was maintained ~300 K using Anderson thermostat^62^. Each system was first energy minimized for 1000 DMD time units (~50 ps) with a strong heat-exchange coefficient with the virtual heat bath^63^, followed by equilibrium simulations carried out for six millions DMD time units, which corresponded to a simulation time of ~300 ns.

### Analysis methods

Secondary structure analyses were performed using the dictionary secondary structure of protein (DSSP) method^64^. A hydrogen bond was considered to be formed if the distance between backbone N and O atoms was ≤3.5 Å and the angle of N−H···O ≥120°^65^. Two chains were considered to form a β-sheet when two or more consecutive residues in each chain adopted the β-strand conformation and these residues were connected by at least two backbone hydrogen bonds. The anti-parallel/parallel β-strand ratio was determined by the number of hydrogen bonds between any two adjacent β-strands forming anti-parallel/parallel β-sheets.

The size of a β-sheet was the number of β-strand in a β-sheet layer. The β-sheet length was determined by the number of continuous residues adopting β-sheet conformations in a given chain. The mass weighted β-sheet size,${\bar{n}}_{\beta -sheet-size}$, was determined by the following equation

1 $${\bar{n}}_{\beta -\mathrm{sheet}-\mathrm{size}}=(\sum _{i=1}^{{n}_{\beta }}{n}_{i}^{2})\div(\sum _{i=1}^{{n}_{\beta }}{n}_{i}),$$

where *n*_*β*_ denoted the number of β-sheets, and *n*_*i*_ was the size of the *ith* β-sheet.

Two peptides inter-connected by at least one inter-molecular heavy atom contact (the cutoff of 0.55 nm) were defined to belong to *an oligomer*. The number of peptides in an oligomer was referred to the oligomer size. A *β-sheet oligomer* was defined as multiple β-sheets inter-connected by at least one heavy atom contact, and the total number of peptides in β-sheet conformation within the complex corresponded to the β-sheet oligomer size. Two peptides in β-sheet conformation (determined by DSSP) formed a β-sheet if they had at least two inter-peptide hydrogen bonds between backbones. If a β-sheet had a closed form with every β-strand in the β-sheet having at least two neighboring β-strands, we defined it as *a β-barrel oligomer*. We used a network-based approach^40^ to automatically detect these β-barrels along the simulation trajectories.

The two-dimensional potential of mean force (PMF, or the effective free energy) was computed according to

2 $$PMF={\textstyle \text{-}}{{\rm{K}}}_{{\rm{B}}}{\rm{T}}\,ln\,P({n}_{oligomer},{n}_{\beta -sheet}),$$

where *K*_*B*_ was the Boltzmann constant, T corresponded to the simulation temperature 300 K, and *P*(*n*_*oligomer*_, *n*_*β-sheet*_) was the probability of an oligomer with the oligomer size *n*_*oligomer*_ and the average number of residues adopting β-sheet conformation per chain, *n*_*β-sheet*_.

## Electronic supplementary material

Supplementary Information
