# Supplementary material for: β-barrel Oligomers as Common Intermediates of Peptides Self-Assembling into Cross-β Aggregates
Source: Sci Rep. 2018 Jul 9;8:10353. doi: 10.1038/s41598-018-28649-7 (PMC6037789; doi:10.1038/s41598-018-28649-7)
Supplement: Supplementary file 1 — Supplementary Information [file 41598_2018_28649_MOESM1_ESM.pdf]

Supplementary Information

β-barrel Oligomers as Common Intermediates of Peptides Self-Assembling into Cross-β Aggregates

Yunxiang Sun, Xinwei Ge, Yanting Xing, Bo Wang, Feng Ding*
Department of Physics and Astronomy, Clemson University, Clemson, SC 29634, USA

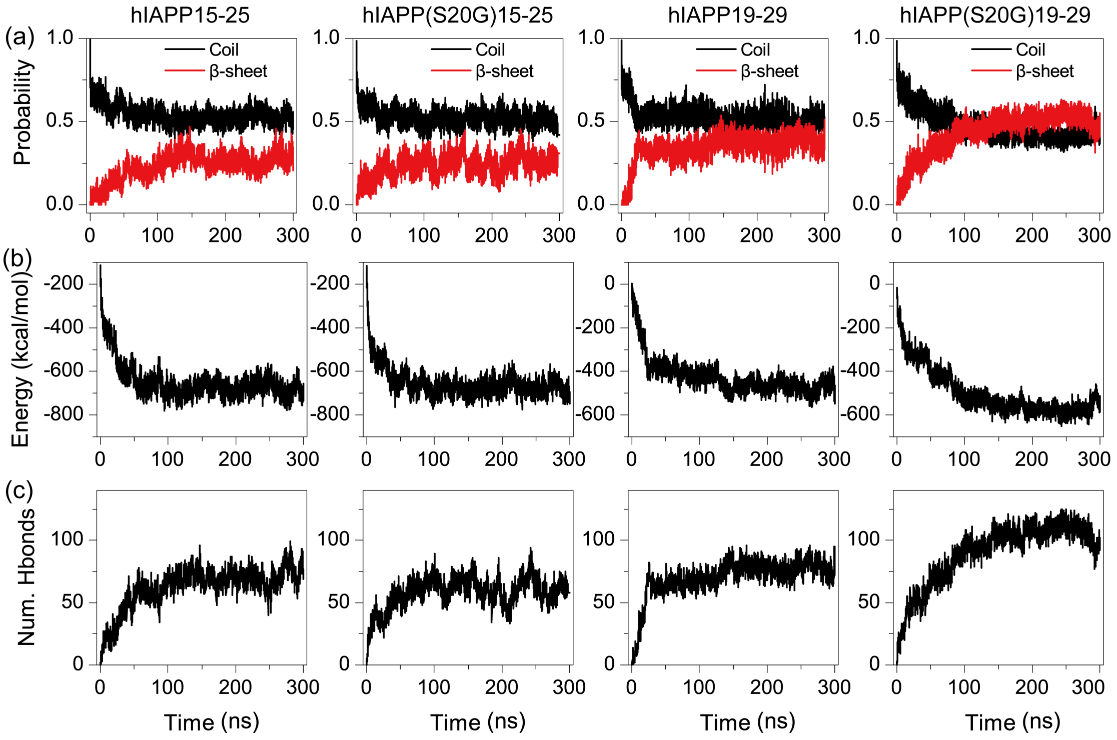

Figure S1. Typical DMD simulation trajectories in terms of (a) coil and β-sheet contents, (b) potential energy and (c) the total number of inter-peptide backbone hydrogen bonds. For illustration purpose, we only showed one out of 10 independent DMD simulation trajectories of the largest molecular systems (i.e., 20 peptides) for IAPP15-25, IAPP(S20G)15-25, IAPP19-29, and IAPP(S20G)19-29. We found that even for the largest molecular systems these structural and energetic parameters reached the apparent steady states after 150 ns.

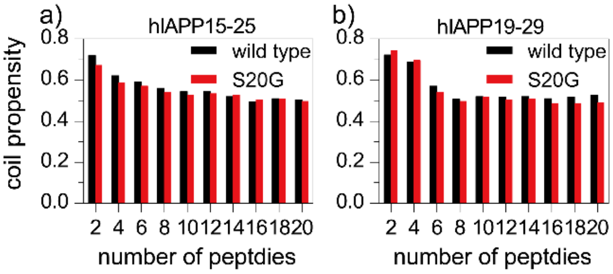

Figure S2. The averaged random coil content in simulations with increasing number of peptides for each of the four sequences of IAPP15-25, IAPP(S20G)15-25, IAPP19-29, IAPP(S20G)19-29. For each molecular system, the last 150 ns simulations of ten independent runs were used for secondary structure analysis.

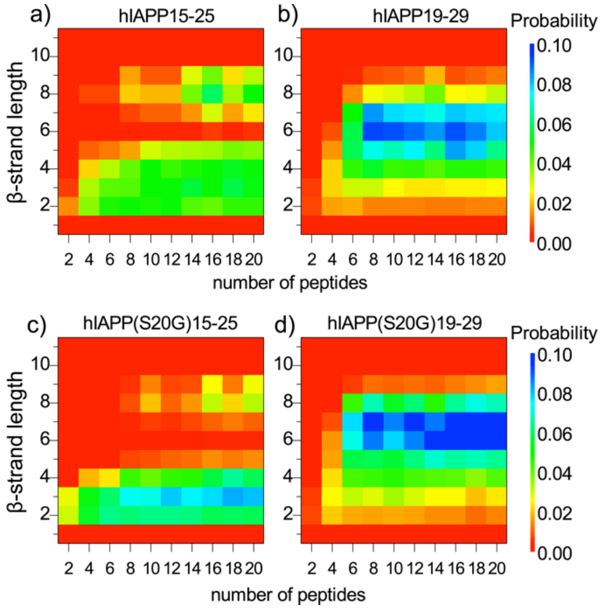

Figure S3. The probability distributions of the β-strand lengths in simulations of (a) hIAPP15-25, (b) hIAPP19-29, (c) hIAPP(S20G)15-25 and (d) hIAPP(S20G)19-29 with different number of peptides .

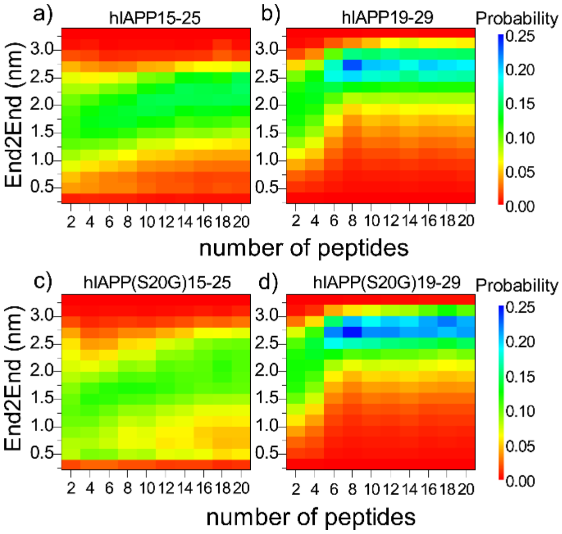

Figure S4. The probability distribution of the end-to-end distances of each peptide simulations of (a) hIAPP15-25, (b) hIAPP19-29, (c) hIAPP(S20G)15-25 and (d) hIAPP(S20G)19-29 with different number of peptides.

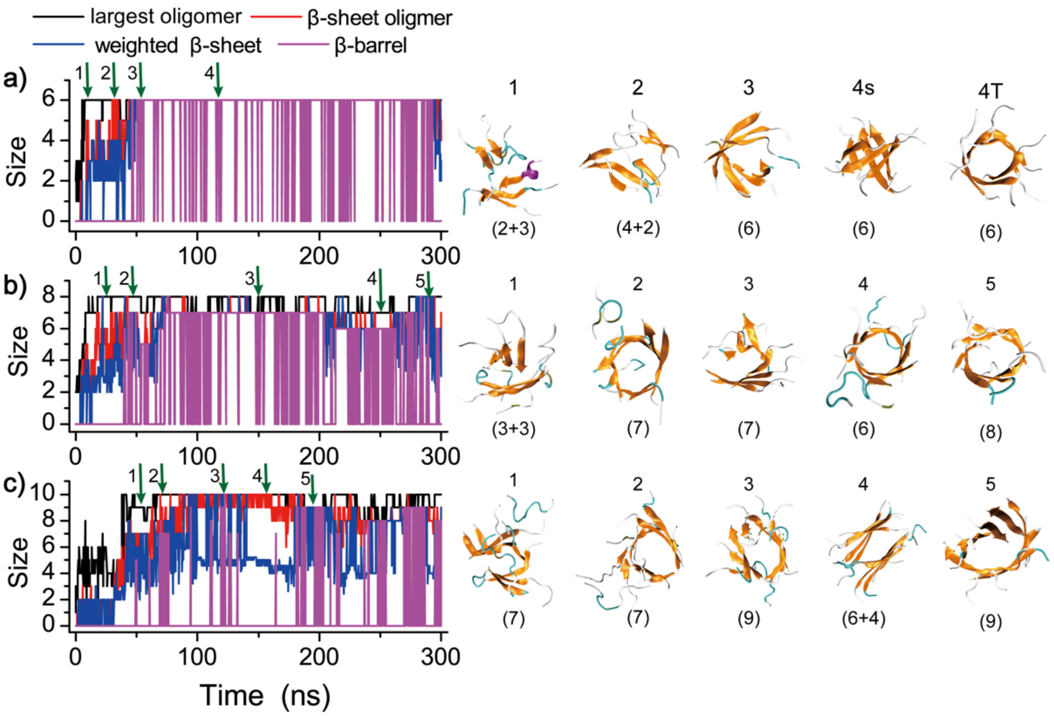

Figure S5. The aggregation dynamics of hIAPP(S20G)19-29. The self-assembly dynamic of (a) six, (b) eight and (c) ten hIAPP(S20G)19-29 peptides were monitored by the time evolution of the largest oligomer size (black), the largest β-sheet oligomer size (red), the mass-weight average β-sheet size (blue) and the total β-barrel size (purple) in each representative trajectory. The snapshot structures along the simulation trajectories as indicated by green arrows were presented on the right lane. Each peptide was shown in cartoon representation with strand colored in yellow, coil in gray, and turn in cyan. The sizes of β-sheets were given in the

parentheses.

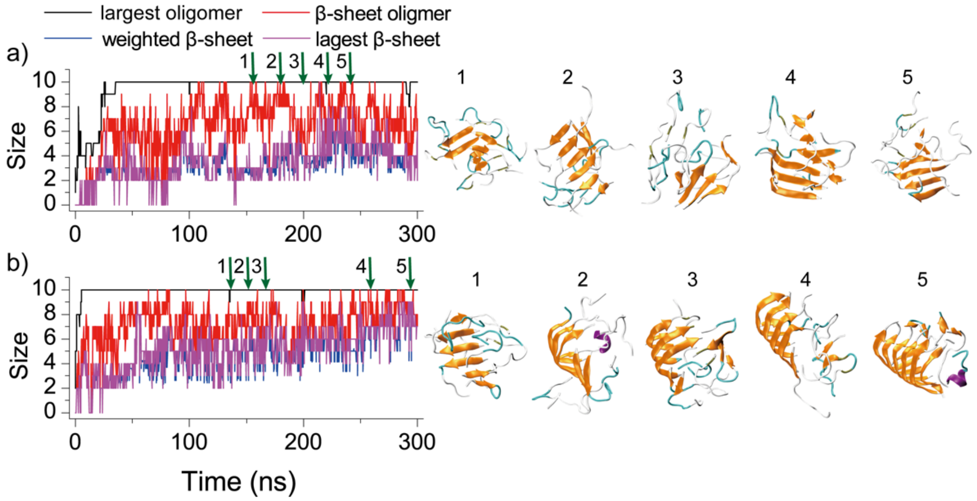

Figure S6. The aggregation dynamics of hIAPP(S20G)15-25. The aggregation processes of ten peptides from isolated random coil conformations to single-layer β-sheets with either L-turn (a) and U-turn (b) morphologies. The largest oligomer size (black), the largest β-sheet oligomer size (red), the mass-weighted average β-sheet size (blue) and the largest β-sheet size (purple) were plotted as the function of simulation time. The snapshot structures along the simulation trajectories as pointed by green arrows were given on the right lane, where each peptide was shown in cartoon representation with strand colored in yellow, coil in gray, helix in purple, and turn in cyan. The sizes of β-sheets were given in the parentheses.

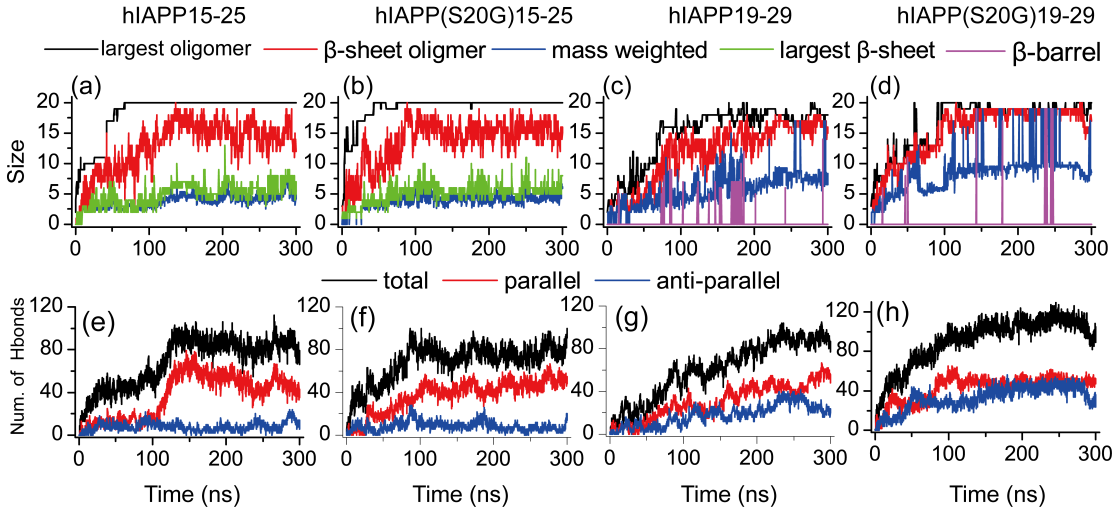

Figure S7. The aggregation dynamics of hIAPP15-25, hIAPP(S20G)15-25, hIAP19-29 and hIAPP(S20G)19-29 in large simulations of 20 peptides. The time evolution of the largest oligomer size, largest β-sheet oligomer size, mass-weighted β-sheet size were colored by black, red and blue, respectively in panels (a-d). The largest β-sheet size was also shown as the green line for (a) hIAPP15-25 and (b) hIAPP(S20G)15-25, and the β-barrel size was shown as the purple line for (c) hIAPP19-29 and (d) hIAPP(S20G)19-29. For each of the trajectories, we also presented the time evolution of the total number of hydrogen bonds (black), within which the

numbers of hydrogen bonds in parallel (red) and anti-parallel (blue) β-sheets were also presented.

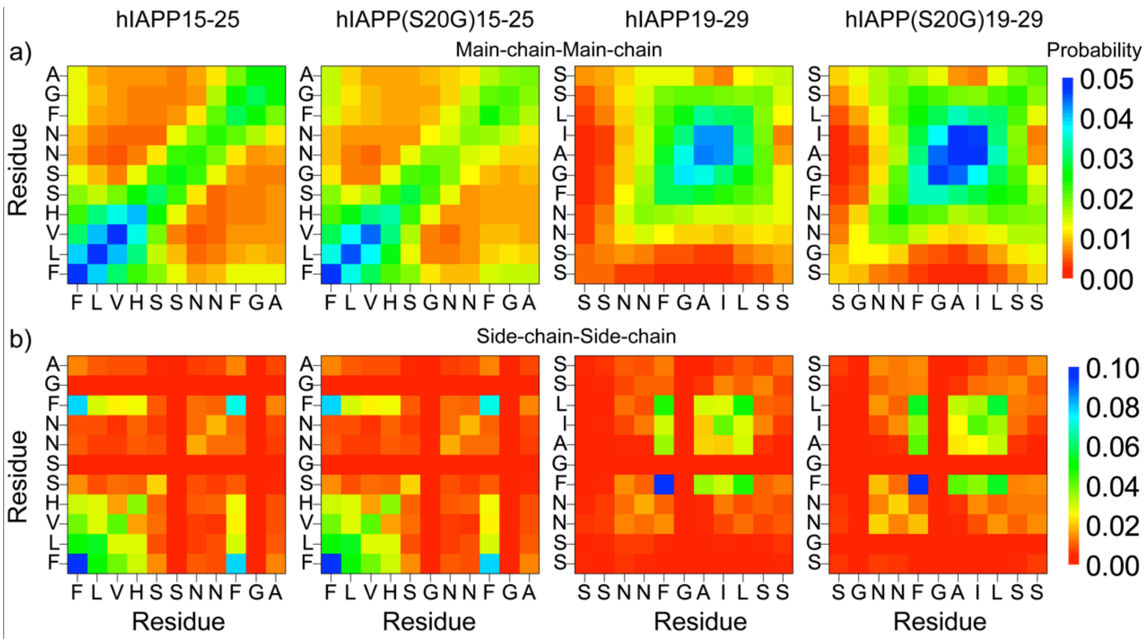

Figure S8. The residue-wise inter-peptide contact frequency maps were computed based on either (a) the backbone-backbone or (b) the sidechain-sidechain interactions for DMD simulations with 20 peptides

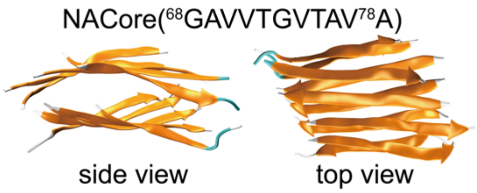

Figure S9. The typical cross-β aggregate structure of amyloidogenic core segment of α-synuclein peptide (NACore). The peptides are shown in the cartoon representation and both side (left) and top (right) views are presented. This aggregate was formed in DMD simulations of ten peptides starting from isolated peptides in the random coil conformation.
